# Supplementary figures and images for: Metamorphic gene regulation programs in Xenopus tropicalis tadpole brain
Source: PLoS One. 2023 Jun 29;18(6):e0287858. doi: 10.1371/journal.pone.0287858 (PMC10310023; doi:10.1371/journal.pone.0287858)

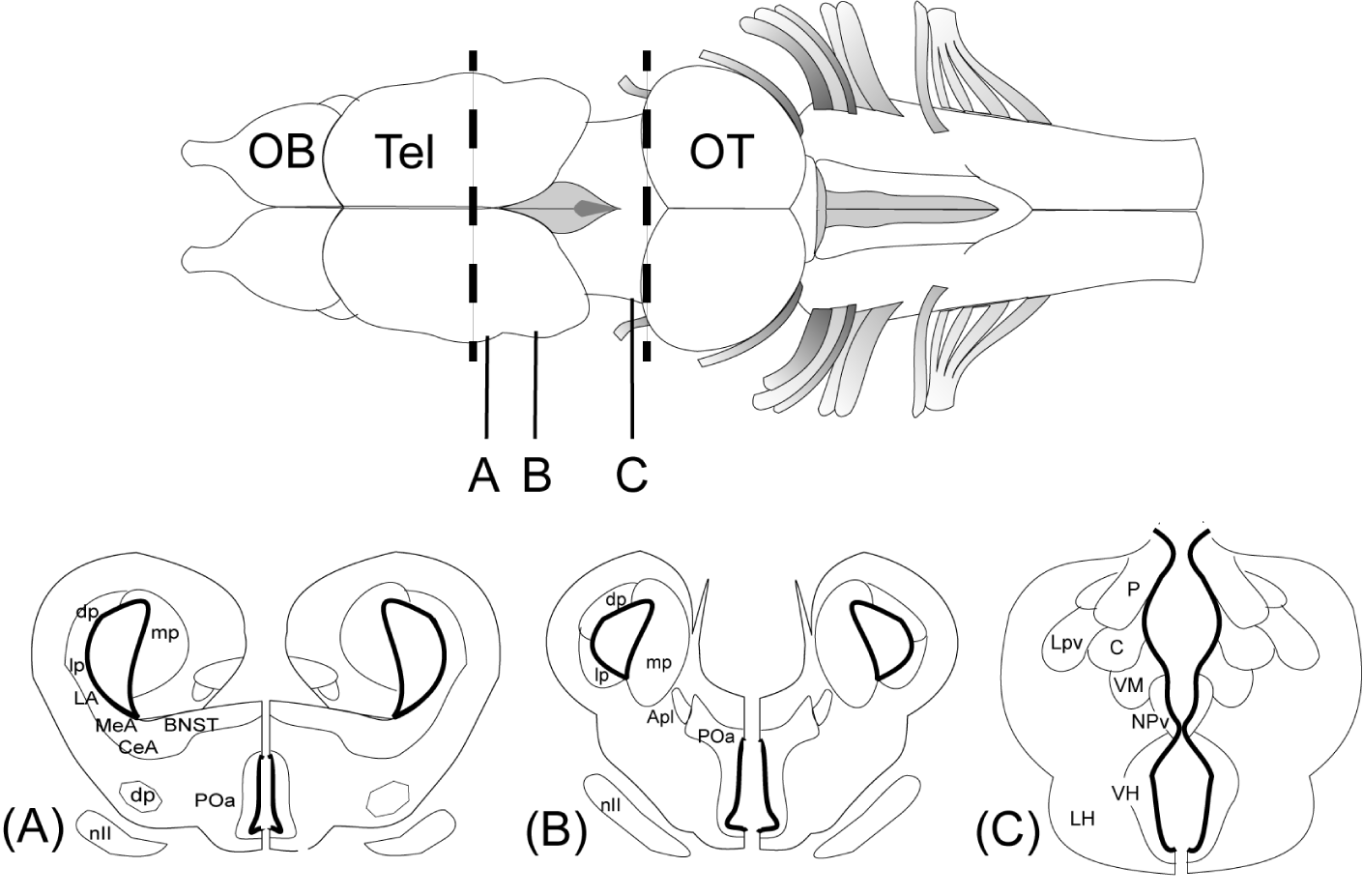

Supplement: S1 Fig — The coronal anatomical diagrams of Xenopus brain are from Tuinhof and colleagues [93] with modifications by Yao and colleagues [94]. Dotted lines demarcate the region of the tadpole brain that was dissected. Abbreviations: Apl, Amygdala pars lateralis; BNST, bed nucleus of the stria terminalis; C, central thalamic nucleus; CeA, central amygdala; dp, dorsal pallium; LA, lateral amygdala; LH, Lateral hypothalamus lp, lateral pallium; Lpv, lateral thalamic nucleus, pars posteroventralis; MeA, medial amygdala; mp, medial pallium; NPv, nucleus of the paraventricular organ; nII, cranial nerve II; OB, olfactory bulb; OT, optic tectum; P, posterior thalamic nucleus; POa, preoptic area; Tel, telencephalon; VH, ventral hypothalamic nucleus; VM, ventromedial thalamic nucleus. (TIF) [file pone.0287858.s001.tif]

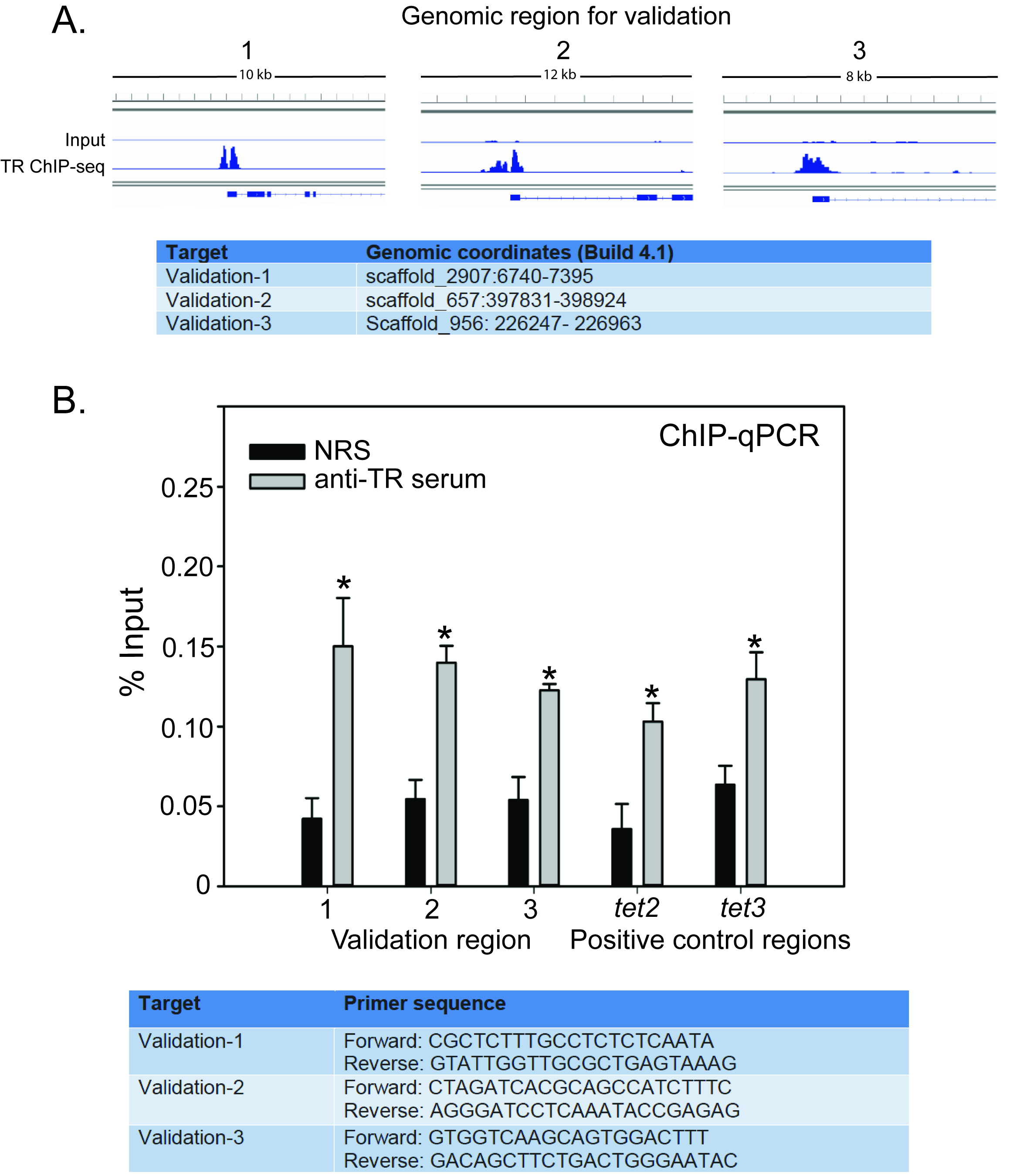

Supplement: S2 Fig — A. Genome browser traces showing the location of TR peaks at three uncharacterized loci. The genomic coordinates are given in the table below the image (1 –XLOC_019343.1; 2 –XLOC_036006.1; 3 –XLOC_032902.1). Bars below the traces indicate the predicted exons and lines the predicted introns. B. Targeted ChIPqPCR assays for TR at the genomic locations shown in panel A using chromatin isolated from brains of tadpoles at metamorphic climax (NF stage 62). The oligonucleotide primers used are shown in the table below the graph. Previously characterized TREs at the tet2 and tet3 loci were included as controls [32]. Chromatin extracts were precipitated using anti-TR serum or normal rabbit serum (NRS) as a control. Bars represent the mean+SEM of the ChIP signal expressed as a percentage of the input (n-4/treatment). Asterisks indicate statistically significant differences between the anti-TR serum and NRS (p<0.05; Student’s t-test). (TIF) [file pone.0287858.s002.tif]

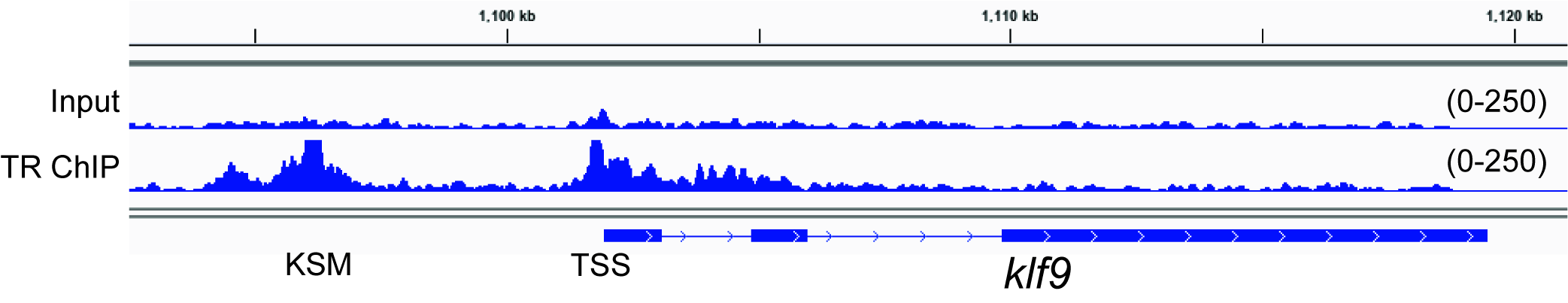

Supplement: S3 Fig — Shown are Integrative Genome Viewer (IGV) genome browser tracks for TR ChIP-seq reads mapped to the Xenopus tropicalis genome. We conducted a TR ChIP-seq experiment on chromatin isolated from the region of the preoptic area/thalamus/hypothalamus of metamorphic climax stage (NF stage 62) X. tropicalis tadpole brain. The input tracks are shown above the TR ChIP-seq tracks. Numbers in parentheses represent the scale for peak height. The gene structures are shown below the genome traces; lines and black filled bars represent introns and exons, respectively, and arrows indicate the direction 5’ → 3’. In addition to the previously discovered TR association at the upstream klf9 synergy module (KSM; which contains a DR+4 T3 response element located ∼6 kb upstream of the transcription start site) [39] we observed TR ChIP-seq peaks 5’ to the KSM, near the transcription start site (TSS) and within the gene. These additional sites of TR association may represent previously uncharacterized thyroid hormone response elements, or perhaps apparent TR association at this region caused by chromosomal looping [39]. (TIF) [file pone.0287858.s003.tif]
