# Supplementary material for: Metamorphic gene regulation programs in Xenopus tropicalis tadpole brain
Source: PLoS One. 2023 Jun 29;18(6):e0287858. doi: 10.1371/journal.pone.0287858 (PMC10310023; doi:10.1371/journal.pone.0287858)
Supplement: S7 Table — Using HOMER software we found that the direct repeat with 4 base spacer (DR4) THRa NR and THRb NR motifs were enriched in the DNA sequences within the TR ChIP-seq peaks. Note that the HOMER software did not show the first nucleotide present in the first 6-base half-site for the THRa and THRb motifs. (DOCX) [file pone.0287858.s010.docx]

**Supplemental Table 7. Direct repeat +4 thyroid hormone response element motifs are enriched in the TR ChIP-seq peaks in tadpole brain.**

| **Motif** | **Name** | **p-value** | **Target sequences with motif** | **% target sequences with motif** |
| --- | --- | --- | --- | --- |
| 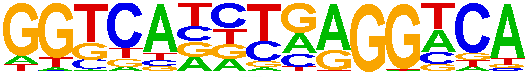 | THRa(NR) | 1.0e-8 | 199 | 3.16% |
| 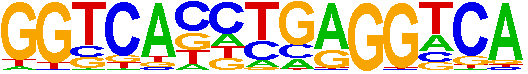 | THRb(NR) | 1.0e-7 | 252 | 4.00% |

Using HOMER software we found that the direct repeat with 4 base spacer (DR4) THRa NR and THRb NR motifs were enriched in the DNA sequences within the TR ChIP-seq peaks. Note that the HOMER software did not show the first nucleotide present in the first 6-base half-site for the THRa and THRb motifs.
